# Supplementary material for: Public discourse and sentiment during the COVID 19 pandemic: Using Latent Dirichlet Allocation for topic modeling on Twitter
Source: PLoS One. 2020 Sep 25;15(9):e0239441. doi: 10.1371/journal.pone.0239441 (PMC7518625; doi:10.1371/journal.pone.0239441)
Supplement: S1 Table — (DOCX) [file pone.0239441.s001.docx]

**S1 Table Hashtags used as key search terms**

| Hashtags used as key search terms | #Coronaoutbreak, #CoronavirusChina, #Wuhan, #Coronavirus, #ChinaCoronavirus, #Wuhan #WuhanCoronavirus, #Wuhanoutbreak, #ChinaVirus, #2019nCoV, #ChineseDon'tComeToJapan, #NoSoyUnVirus, #IamNotVirus, #JeNeSuisPasUnVirus, #Xenophobia, #PrayForChina, #DrLiWenLiang, #ItWillGetBetter, #BeStrongChina. |
| --- | --- |
