# Supplementary material for: Public discourse and sentiment during the COVID 19 pandemic: Using Latent Dirichlet Allocation for topic modeling on Twitter
Source: PLoS One. 2020 Sep 25;15(9):e0239441. doi: 10.1371/journal.pone.0239441 (PMC7518625; doi:10.1371/journal.pone.0239441)
Supplement: S1 File — (DOCX) [file pone.0239441.s002.docx]

**Data Availability statement**

**The present study used data collected from a Third-party – Twitter. Based on Twitter’s Developer’s terms and policy, we have restrictions to redistribute Twitter content to third parties, as follows,**

If you provide Twitter Content to third parties, including downloadable datasets or via an API, you may only distribute Tweet IDs, Direct Message IDs, and/or User IDs (except as described below). We also grant special permissions to academic researchers sharing Tweet IDs and User IDs for non-commercial research purposes.

In total, you may not distribute more than 1,500,000 Tweet IDs to any entity (inclusive of multiple individuals associated with a single entity) within any 30 day period unless you have received written permission from Twitter. In addition, all developers may provide up to 50,000 public Tweets Objects and/or User Objects to each person who uses your service on a daily basis if this is done via non-automated means (e.g., download of spreadsheets or PDFs).

Academic researchers are permitted to distribute an unlimited number of Tweet IDs and/or User IDs if they are doing so on behalf of an academic institution and for the sole purpose of non-commercial research. For example, you are permitted to share an unlimited number of Tweet IDs for the purpose of enabling peer review or validation of your research. If you have questions about whether your use case qualifies under this category please submit a request via the [API Policy Support form](https://help.twitter.com/forms/platform).

Any Twitter Content provided to third parties remains subject to this Policy, and those third parties must agree to the Twitter [Terms of Service](https://twitter.com/en/tos), [Privacy Policy](https://twitter.com/en/privacy), [Developer Agreement](https://developer.twitter.com/en/developer-terms/agreement), and [Developer Policy](https://developer.twitter.com/en/developer-terms/policy) before receiving such downloads. You may not enable any entity to circumvent any other limitations or restrictions on the distribution of Twitter Content as contained in this [Policy](https://developer.twitter.com/en/developer-terms/policy), the [Developer Agreement](https://developer.twitter.com/en/developer-terms/agreement), or any other agreement with Twitter.

**Source:** <https://developer.twitter.com/en/developer-terms/policy>

**Thus, we indicate that potential interested researchers can contact us upon their unique requests for sharing the data. We will follow strictly to the Twitter’s terms and policy. We also indicated it in the manuscript.**
